# Pain assessment and management: An audit of practice at a tertiary hospital

**DOI:** 10.4102/hsag.v25i0.1281

**Published:** 2020-01-20

**Authors:** Agya B.A. Prempeh, Rowan Duys, Alma de Vaal, Romy Parker

**Affiliations:** 1 Department of Anaesthesia and Perioperative Medicine, Groote Schuur Hospital, University of Cape Town, Cape Town, South Africa

**Keywords:** pain, assessment, management, perioperative, South Africa

## Abstract

**Background:**

In spite of advances in techniques and analgesics for pain management, pain remains a major health problem. Regular assessment and reassessment of pain using guidelines with measurable goals is essential for effective pain management in surgical wards. Unfortunately, no such guidelines exist in South Africa. To implement appropriate precepts for the South African context, the current practice must be understood.

**Aim:**

The aim of this article was to evaluate pain assessment and management of patients in two surgical wards at a tertiary hospital in South Africa.

**Setting:**

The study was conducted within the Western Cape Province of South Africa in a government-funded tertiary academic institution. The patients at this hospital are generally from the low-income strata and live in resource-poor communities.

**Methods:**

A cross-sectional, retrospective medical record audit was conducted. The folders of all 215 patients admitted to a specific orthopaedic trauma and urogynaecological ward of a tertiary hospital in South Africa over a span of 1 month were targeted for review. Medical folders that were not available or had missing notes were excluded. Variables evaluated included the number of pain assessments recorded, pain assessor, assessment tool and management plan.

**Results:**

A total of 168 folders were available for review. Nearly half of the patients had no documented pain assessment. The Verbal Rating Scale was the predominant tool used, and assessments were mostly conducted by the ward doctor. Pain interventions appeared to be primarily based on the professional knowledge of the practitioner and were not evidence-based.

**Conclusion:**

Pain assessment and management was a problem in the two wards reviewed, which is similar to the findings from studies referenced in this text. Health professionals must be empowered to manage pain adequately. An assessment tool that integrates the biopsychosocial factors that influence the pain experience should be routinely employed by a multidisciplinary team to facilitate goal-directed therapy.

## Introduction

Pain is a subjective phenomenon defined by the International Association for the Study of Pain as an ‘unpleasant sensory and emotional experience associated with actual or potential tissue damage or described in terms of such damage’ (Merskey & Bogduk 1994). It can be classified based on its time course as either acute or chronic. Acute pain has an abrupt onset and may last up to 6 months if poorly managed. Thereafter, it evolves into chronic pain via maladaptive neuroplasticity. Inadequate management of pain results in undesired outcomes, including poor patient satisfaction, impaired immunity, delayed wound healing, prolonged hospital stay and increased hospital costs (Wuhrman & Cooney 2011). In spite of advances in pain management techniques and analgesics, it is estimated that one in five adults suffer from pain with the predominant causes being trauma, inflammation, neoplasm and circulatory changes. Of the patients in the surgical trauma subgroup, more than 80% experience acute pain in the postoperative period with nearly three-quarters describing their pain as significant, that is, moderate, severe or extreme (Apfelbaum et al. 2003).

There are limited data on acute pain assessment in the hospital setting. In a study at the Clinical Centre of Vojvodina, 77% of the 135 inpatients interviewed on the second postoperative day either ‘disagreed’ or ‘strongly disagreed’ on whether their pain intensity was regularly assessed by the health professionals in the ward (Milutinović et al. 2009). Another study at a district general hospital in West Norfolk reported that assessment of pain among 140 geriatric patients was substandard. Only two-thirds of the patients had a documented pain assessment within the first 24 h of their hospital admission (Niruban et al. 2010). In South Africa, there also appears to be a paucity of reporting on pain assessment. A small study involving 12 registered nurses working in a South African tertiary academic hospital reported that the nurses did not have a standard approach for assessing pain. In particular, none of them utilised a pain rating scale; the nurses based their pain assessment on ‘how the patient looked’, ‘what the patient said’, ‘the patient’s way of talking’ and the nurses’ experience of similar circumstances (Klopper et al. 2006). This method of pain assessment has questionable validity and limited application.

Given the subjectivity of pain, the gold standard for its assessment is a validated self-report tool. Where self-report is not possible, such as with communication difficulties, behavioural assessment tools assessing vocalisation, facial grimacing and restlessness are indicated (Gregory & Richardson 2014). The most commonly used self-report tools for evaluating pain intensity in an acute setting are the Likert-type numeric rating and the visual analogue scales (Myles et al. 2017). The numeric rating scale assesses the intensity of pain using a grading system from 0 (no pain) to 10 (worst imaginable pain). The visual analogue tool is a 100 mm continuous scale with verbal descriptors along its length corresponding to numeric values. Respondents specify the point along the scale, which best represents their perceived pain status. The McGill Pain Questionnaire, on the other hand, evaluates both the quality and the intensity of the pain experienced (Melzack 2005). This questionnaire comprises four categories, with 78 words in total. Respondents choose the words that best depict their pain experience. The categories include pain descriptors, affective components of pain, evaluation of pain and a miscellaneous group. To quantify the intensity of pain using this tool, a numerical value is assigned to each chosen word. The summed-up score of the numerical values reflects the respondent’s pain intensity. Scores range from 0 (no pain) to 78 (severe pain). Other validated assessment tools include the Verbal Rating Scale (mild, moderate and severe), Wong-Baker Faces Pain Rating Scale and the Pain Quality Assessment Scale (Gregory & Richardson 2014). Currently, none of the validated assessment tools addresses all three biopsychosocial facets of pain, namely, intensity, cognitive function and impact on functional activity.

Several recommendations have been made regarding the routine assessment of pain in the hospital setting (Chou et al. 2016). The frequency of pain assessments and reassessments may be guided by the type of surgery performed, comorbidities present and the pharmacodynamics of the analgesic agent, that is, the onset of action and the time to reach peak effect. For physical therapy, the onset of relief from pain often occurs during or soon after the intervention (Chou et al. 2016). Therefore, the optimal timing of pain assessment and reassessment is variable, with a lack of strong evidence to support specific regimens. Despite the apparent uncertainty of optimal timing, which will be dependent on the pain management strategy utilised, the recommendations that pain should be assessed and reassessed regularly to monitor the effectiveness of management are consistent (Chou et al. 2016).

The effective management of pain requires a good understanding of the mechanisms of pain and the consequences of poorly managed pain, among other factors (Sinatra 2010). When health professionals have in-depth knowledge, it is manifested in their choice and application of analgesics (Nuseir, Kassab & Almomani 2016). There is substantial evidence that multimodal analgesia or a combination of pharmacological agents and non-pharmacological methods should be used to manage pain (Chou et al. 2016). This approach is associated with improved pain scores and a reduction in opioid use as compared with the empirical pain management method that is predominantly based on the professional knowledge of the practitioner. An example of a multimodal analgesia approach is the World Health Organization (WHO) analgesic ladder (Cura Della Redazione 2016). In this conceptual ladder, analgesics are adjusted in a stepwise manner from non-opioids through to potent opioids (step-up) or vice-versa (step-down), consistent with the patient’s reported pain intensity. Non-pharmacological adjuncts such as transcutaneous electrical nerve stimulation (TENS) can be considered although the evidence-based recommendation for its use is weak (Chou et al. 2016). Other non-pharmacological interventions such as acupuncture, cold therapy and massage lack sufficient evidence to be recommended or discouraged from being used (Chou et al. 2016). However, to reduce exposure to the unpleasant side effects of pharmacological and non-pharmacological pain interventions, pain management should be goal-directed and individualised based on the patient’s biopsychosocial characteristics and the extent of trauma. Goal-directed therapy emphasises functional well-being, as opposed to total pain relief. Functional restoration aims to improve the quality of life by promoting deep breathing, coughing, mobilisation out of bed, reducing stress, enhancing sleep patterns, and boosting family and social relationships (Tseng et al. 2014).

In spite of the wide range of evidence-based pain management options, optimal postsurgical pain management remains a challenge in a variety of settings (Sinatra 2010). In a systematic review of 165 studies on acute pain after major surgery, the overall mean incidences of moderate to severe pain and severe pain in the first 24 h after surgery were 30% and 11%, respectively. Variation was observed in pain scores depending on the analgesic technique employed. Lower scores were seen in the patient groups that received patient-controlled and epidural analgesia, compared with those who received intramuscular analgesia (Dolin, Cashman & Bland 2002). A German prospective cohort study of 50 523 participants reported that patients had high pain scores on the first postoperative day after common minor surgical procedures, such as appendicectomy and haemorrhoidectomy, suggesting that pain management was inadequate (Gerbershagen et al. 2013). According to WHO data, more than 80% of the global population has no access to treatment modalities for moderate to severe pain (Morriss & Roques 2018).

In low- and middle-income countries, because of resource constraints, there are insufficient data on the incidence and impact of inadequate postsurgical pain management in the current literature (Walters et al. 2016). The majority of surgical procedures performed include caesarean sections and hernia repairs. These procedures are known to result in high rates of chronic postsurgical pain (Morriss & Roques 2018). In a study at a tertiary academic hospital in South Africa, 62% of the 1231 patients who had surgery during office hours retrospectively described their worst pain experienced as moderate or severe. Procedures with the highest incidence of pain included caesarean sections and lower limb orthopaedic surgeries (>80%). In a subgroup of 577 patients managed with oral and intramuscular analgesics in the postoperative period, those experiencing moderate or severe pain received only 46% of the prescribed dose of morphine (Murray & Retief 2016). These findings suggest that there may be problems with pain management in South African hospitals, particularly in patients undergoing surgical procedures.

This study aimed to evaluate pain assessment and management practices in two surgical wards at a tertiary hospital in South Africa over a span of 1 month. Findings will be used to facilitate the implementation of guidelines that are goal-directed and individualised based on the patient’s biopsychosocial characteristics and the extent of trauma, and to strengthen the ongoing training of health professionals in pain assessment and management.

## Methods

A cross-sectional, retrospective medical record audit was conducted. The folders of all 215 patients admitted to specific orthopaedic trauma and urogynaecological wards of a tertiary hospital in South Africa over a span of 1 month were targeted for review. These wards cater to a large number of patients undergoing intermediate to major surgery and thus have high analgesic requirements. Medical folders that were not available or had missing notes were excluded. The folders were requested from the Medical Records Department and reviewed by four investigators. To standardise the data-collection process, a fundamental assumption was made – whatever was not documented was not done.

The variables observed in this audit were: age, gender, admission ward, cognitive disability, functional impairment, native language, type and number of surgeries undertaken, number of pain assessments recorded, pain assessor, assessment tool, pain management plan and delivery, and any reassessment conducted after the planned intervention. The pain assessment tools of interest were the Verbal Rating Scale (mild, moderate and severe), Wong-Baker Faces Pain Rating Scale, Numeric Rating Scale, Visual Analogue Scale, McGill Pain Questionnaire and Pain Quality Assessment Scale (Gregory & Richardson 2014). A pain management plan was defined as being present if there was any written instruction for analgesics or non-pharmacological pain interventions in the patient’s notes or the documentation of analgesics on the prescription chart. An empirical management approach was defined as any management strategy that appeared to be based on the professional knowledge of the practitioner and not supported by evidence-based guidelines of pain management.

No information revealing the identity of a patient was given. The data were entered into a secure database that was accessible only to the investigators, summarised and presented as means and standard deviations (SD) with ranges for completeness. Associations between pain assessment and variables, such as gender, age group, native language, cognitive impairment and admission ward, were determined using Pearson’s chi-squared test. Significance was accepted at *p* < 0.05 throughout.

## Ethical considerations

This audit was granted ethical approval by the University of Cape Town, Faculty of Health Sciences, Human Research Ethics Committee (HREC Ref: 586/2015), and the Groote Schuur Hospital Ethics Board. The principles of the Declaration of Helsinki were adhered to throughout (Millum, Wendler & Emanuel 2013).

## Results

### Patient characteristics

**FIGURE 1:**
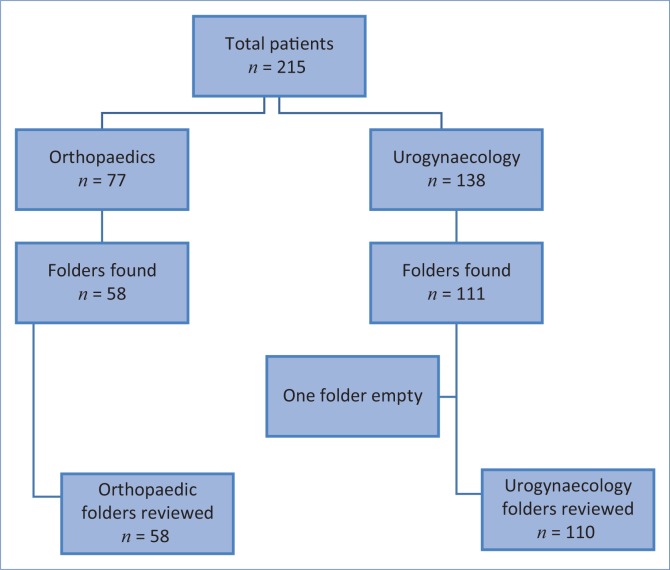
Flow diagram of the data-collection process.

The medical records office traced 169 of the 215 folders that were targeted, one of which had no notes; 168 folders were therefore available for audit (Figure 1). The mean age of the patients was 38 years (15–91, SD 17). For the patients in the urogynaecology ward, it was 36 years (16–83, SD 14), while in the orthopaedic ward it was 42 years (15–91; SD 21). The urogynaecology patients were all female (110), while in the orthopaedic ward there were 21 female and 37 male patients. The most common native language was English (83), followed by isiXhosa (41), Afrikaans (38), Northern Sotho (1) and Setswana (1). Four patients were not citizens of South Africa; their native language was classified as ‘other’. Seven of the patients had been diagnosed with a cognitive disability (five in orthopaedics, two in urogynaecology), and each of these patients had an associated functional impairment. Three had attention deficit disorder, two had memory impairment, one had an impairment in linguistic comprehension and one had an undisclosed functional impairment (Table 1).

**TABLE 1:** Patient characteristics.

Variable age (years)	Number	%
< 20	11	7
20–39	98	58
40–59	39	23
60–79	13	8
> 80	7	4
**Gender**		
Female	131	78
Male	37	22
**Native language**		
English	83	49
isiXhosa	41	24
Afrikaans	38	23
Other	4	2
Northern Sotho	1	1
Setswana	1	1
**Cognitive disability**		
No	161	96
Yes	7	4
**Functional impairment**		
Nil	161	96
Attention	3	2
Memory	2	1
Linguistic comprehension	1	0.5
Other	1	0.5

**TABLE 2:** Surgical procedures performed (*n* = 168).

Orthopaedic procedures (*n* = 58)	Number	%	Urogynaecological procedures (*n* = 110)	Number	%
Open reduction and internal fixation	27	46.6	No surgical intervention	38	34.5
Hip arthroplasty	9	15.5	Termination of pregnancy	15	13.6
No surgical intervention	6	10.3	Evacuation of uterus	10	9.1
Tendon repair	5	8.6	Hysteroscopy	10	9.1
Wound debridement	4	6.9	Total abdominal hysterectomy	7	6.4
Soft tissue repair	3	5.2	Cystoscopy	5	4.5
Irrigation of sepsis	2	3.4	Laparotomy	4	3.6
Spinal fusion	1	1.7	Laparoscopy	4	3.6
Reduction of dislocation	1	1.7	Ectopic pregnancy	4	3.6
			Uterine artery embolisation	2	1.8
			Caesarean section	2	1.8
			Gastroscopy	2	1.8
			Rectovaginal fistula repair	1	0.9
			Ovarian cystectomy	1	0.9
			Polypectomy	1	0.9
			Vaginal tear repair	1	0.9
			Bartholin’s abscess drainage	1	0.9
			Myomectomy	1	0.9
			Perineal tear repair	1	0.9

### Pain assessment

**FIGURE 2:**
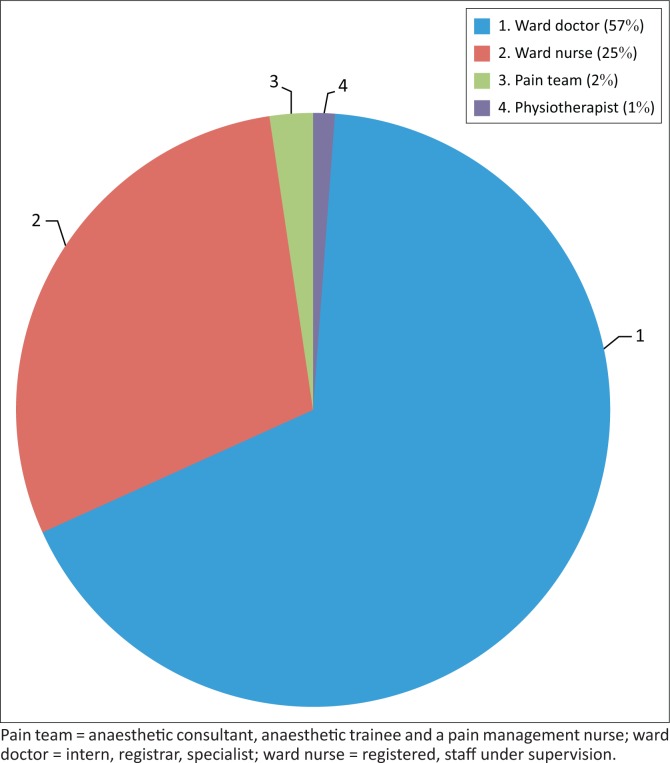
Pain assessments conducted by health professionals (*n* = 85).

Pain was assessed in 85 of the 168 patients (51%) (62 of the 110 urogynaecology patients; 23 of the 58 orthopaedic patients) and reassessed in 55 of the 85 patients (65%) who had received an initial pain assessment. The ward doctor (intern, registrar or specialist) assessed pain in 67% of the 85 patients evaluated (Figure 2). The most common method of pain assessment was the Verbal Rating Scale, which was used for 74 (87%) of the patients assessed.

### Pain management

A pain management plan was documented for 140 of the 168 patients (83%), irrespective of them having a documented pain assessment. Of the 83 patients who had no documented pain assessment, 70% had a pain management plan. Pain management methods included an empirical approach, the WHO analgesic ladder (stepping-up medication, stepping-down medication) and the referral for physiotherapy. The most common method was the empirical approach (see Introduction), which was used in 106 of the 140 patients (76%) (Table 3). A pain management plan was followed in 124 of the 140 patients (89%). Although 44 of the 168 patients had not yet undergone a surgical intervention, 31 (70%) had a documented pain management plan, indicating that they may have been experiencing pain.

**TABLE 3:** Pain management methods used (*n* = 140)†.

Methods	All patients number	%	Orthopaedics number	%	Urogynaecology number	%
Empirical therapy	106	75.7	37	77.1	69	75.0
Step-up medication (medical therapy only)	26	18.6	4	8.3	22	23.9
Step-up medication and physiotherapy (medical and physical therapies)	6	4.3	5	10.4	1	1.1
Step-down medication (medical therapy only)	1	0.7	1	2.1	0	-
Other	1	0.7	1	2.1	0	-

**Total**	**140**	**-**	**48**	**-**	**92**	**-**

† , 140 out of the 168 patients had a documented pain management plan (*n* = 140).

### Relationships between pain assessment and patient characteristics

There was no association between a documented pain assessment and the following variables: gender (*χ*^2^ = 2.29; *p* = 0.13), age group (*χ*^2^ = 2.75; *p* = 0.43), native language (*χ*^2^ = 2.07; *p* = 0.84) and ward of admission (*χ*^2^ = 3.64; *p* = 0.06). The patients with cognitive impairment appeared to have had their pain assessed as often as those without impairment (*χ*^2^ = 1.54; *p* = 0.22).

## Discussion

In this audit of pain assessment and management practice over a period of 1 month in an orthopaedic trauma and urogynaecological ward, 168 folders were reviewed. A total of 140 patients (83%) had a documented pain management plan, and in 89% of these patients, the pain management plan was followed. Of the 140 patients with a pain management plan, 58 (41%) had no evidence of a pain assessment. When a pain assessment was conducted, it was usually documented by the ward doctors. The treatments prescribed were predominantly pharmacological and did not seem to follow evidence-based pain management guidelines (Chou et al. 2016; Cura Della Redazione 2016). Whereas several variables, such as gender, age and native language, have been reported as influencing the likelihood of pain being assessed and managed adequately, in this study, there was no association with the variables tested (Wandner et al. 2012).

Although it is encouraging that a pain management plan was documented and followed in more than 80% of the patients, only 59% of these patients had a pain assessment. This discrepancy between the practices of pain assessment and pain management raises questions about the information on which the clinicians based their treatment decisions. Equally concerning is the question whether clinicians were assessing pain but not documenting it. Poor documentation hinders periodic appraisal of clinical practice and also has medico-legal implications (Chanvej et al. 2004). The lack of agreement between documented assessment and treatment of pain is frequently reported (Chanvej et al. 2004). In a Thai audit of 424 hospital charts, only 0.5% of patients had regular pain assessments documented in the first 24 h, postoperatively. In the first three postoperative days, there was also evidence of inconsistent and inadequate documentation of pain assessment and monitoring (Chanvej et al. 2004). Guidelines recommend that pain should be regularly assessed and reassessed, either at the same intervals as vital sign monitoring or at intervals linked to the expected time in which a treatment should take effect (Chou et al. 2016). Validated pain assessment tools, which measure pain intensity or degree of relief and its impact on functional activity, should be used (Chou et al. 2016). In this audit, no record of assessing pain interference with functional activity or quality of life was found.

Methods to address these gaps in the assessment of pain include education and training of health professionals. An audit series at a University Hospital in Uppsala, Sweden, demonstrated the value of regular training and education in improving pain assessment (Karlsten, Ström & Gunningberg 2005). An initial audit at this Swedish hospital found that only 69% (185/270) of the postoperative patients had their pain assessed using a numeric rating scale according to hospital protocols, with only 45% being reassessed. Following an education and training programme for nurses and surgeons, which required recertification every 3 years, improvements in pain assessment documentation were recorded. One year after the implementation of the training programme, these improvements were minimal (from 69% to 72% of postoperative patients having pain assessed). After 2 years, however, there was a significant improvement, with 90% of folders having pain assessment documented, demonstrating that the education and training intervention appeared to be effective.

Pain is tri-dimensional, that is, biological, psychological and social. Thus, a multidisciplinary team is required to address all the factors that affect the pain experience. Multidisciplinary pain management should incorporate medical, psychological and physical therapies (Darnall, Carr & Schatman 2017). At the turn of the century, there was a surge in anaesthesia-based acute pain services to improve outcomes (Werner et al. 2002). While anaesthetic departments drive these services, the team usually comprises a staff anaesthetist, a nurse trained in pain management and a physiotherapist. Although the service can provide high standards of pain care in surgical wards, only a small proportion of patients may benefit because of cost implications (Rawal 1999). ‘Nurse-based anaesthetist-supervised’ acute pain services can broaden coverage with pain assessed in every patient who undergoes surgery (Rawal 1999). This acute pain service model aims for optimal utilisation of pain management resources.

In this audit, there appeared to be poor coordination between the multiple professionals involved in patient care and providing acute pain services. This could be an explanation for the predominance of pharmacological pain interventions. The empirical approach was the most common pain management method used, with no clear basis for prescribing practices. Only 33 of the 140 patients with a documented pain management plan (24%) had analgesics stepped-up or -down as per the WHO analgesic ladder (Cura Della Redazione 2016). Thus, protocols appear to be necessary in the wards included in this audit to facilitate multidisciplinary management of pain using evidence-based approaches. The lack of protocols negatively influences pain management outcomes (Karlsten et al. 2005).

The strengths of a retrospective audit include reporting on real-world clinical practice without biasing clinical documentation. However, it is well-reported that prospective studies of clinical practice change clinician behaviour in the short term and may not be capturing the real-world practice (Thadhani & Tonelli 2006). Furthermore, an audit allows for the identification of a problem within a specific setting and provides baseline data for determining whether interventions to address the identified problem are effective. However, audits are not generalisable as they are limited to the setting – in this case, to two specific wards in a tertiary teaching hospital. Also, the medical folders that were unavailable or had missing notes in conjunction with the group of patients who had not yet undergone a surgical intervention may have impacted the findings of this study. Hence, while the reported results may be relevant elsewhere, they must be considered within the context.

## Conclusion

This audit suggests that pain assessment and management was a problem in the two wards reviewed at the tertiary hospital. This finding is similar to the other studies referenced in this text (Dolin et al. 2002; Gerbershagen et al. 2013; Klopper et al. 2006; Milutinović et al. 2009; Murray & Retief 2016; Niruban et al. 2010). Nearly half of the patients had no documented pain assessment. When a pain assessment was conducted, the ward doctor was at the forefront, departing from the nurse-based acute pain service model, which empowers nurses to manage pain in the wards (Rawal 1999). A significant proportion of the pain interventions appeared to be based on the professional knowledge of the practitioner and not supported by evidence-based guidelines of pain management (Chou et al. 2016; Cura Della Redazione 2016). Pain management is not merely about the reduction of pain; it is also about the optimisation of recovery through a reliable and accurate assessment of pain, which was not demonstrated in this study. Thus, an assessment tool, which integrates the biopsychosocial factors that influence the pain experience, should be employed by a multidisciplinary team to facilitate goal-directed therapy. We recommend that a multidisciplinary education and training programme on pain assessment, management and documentation be implemented using evidence-based protocols, case-based teaching and a multifaceted pain assessment tool. Review audits and studies of patient satisfaction with pain management would provide in-depth information on the efficacy of the training programme. We also suggest conducting of further studies assessing patient and health professionals’ perspective on pain and its management to aid comprehension of this complex phenomenon.
